# Retraction: Pooled prevalence and determinants of modern contraceptive utilization in East Africa: A Multi-country Analysis of recent Demographic and Health Surveys

**DOI:** 10.1371/journal.pone.0344523

**Published:** 2026-03-10

**Authors:** The PLOS One Editors

Following the publication of this article [1], concerns were raised regarding the data and results. Specifically:

- Fig 1 presents cross-sectional, descriptive prevalence data using a funnel plot, a visualization typically associated with meta-analyses, even though the analysis reported in this figure is not a meta-analysis. In addition, the percentage of women using modern contraceptives was incorrectly labeled as an “effect size” in this figure.
- The percentage of women using modern contraceptives in Burundi and Kenya, reported in the Results section and Fig 1, appears to be inconsistent with data from the Demographic and Health Surveys (DHS; https://dhsprogram.com) (variable v364), which the authors state was used for their analysis: 29.78% and 16.16% versus 14.6% and 39.1%, respectively.
- The percentage of women in Comoros using modern contraceptives, reported in the Results section and Fig 1, is about 6-fold more than the percentage reported by FP2030 (Family Planning 2030; a country-led global partnership supported by national governments and UN agencies) for the same year (61.49% versus 10.7% [2]).
- The authors did not provide a clear definition of the population underlying the “total” reported in Fig 1. As a result, it is unclear which, if any, DHS-defined group was used, which limits interpretation of the reported estimates.
- In the Results section and Table 3, the authors reported that, compared with the reference country Burundi, women living in Comoros had lower odds of using modern contraceptives (odds ratios 0.22 and 0.26 in Models II and III, respectively), corresponding to 77% lower odds. This contradicts the data presented in Fig 1, which indicate that 29.78% of women in Burundi use modern contraceptives compared with 61.49% in Comoros.

The corresponding author maintained that all analyses were correct and provided DHS data from Burundi. However, during editorial review inconsistencies were noted between these data and the data reported in [1]. The authors did not respond to subsequent editorial communications regarding these concerns.

A member of the Editorial Board assessed the concerns raised. They agreed with the issues outlined above, and reported that the values presented in Fig 1 differ markedly from the corresponding reference data.

In light of the above concerns, which call into question the validity and reliability of the published results and conclusions, the *PLOS One* Editors retract this article.

All authors either did not respond directly or could not be reached.
