# Correction: Retraction: Pooled prevalence and determinants of modern contraceptive utilization in East Africa: A Multi-country Analysis of recent Demographic and Health Surveys

**DOI:** 10.1371/journal.pone.0345823

**Published:** 2026-03-26

**Authors:** The PLOS One Editors

The first bullet point in this Retraction notice [1] for [2] incorrectly refers to a forest plot as a funnel plot. The correct bullet point is:

- Fig 1 presents cross-sectional, descriptive prevalence data using a forest plot, a visualization typically associated with meta-analyses, even though the analysis reported in this figure is not a meta-analysis. In addition, the percentage of women using modern contraceptives was incorrectly labeled as an “effect size” in this figure.

The publisher apologizes for the error.
